# Effects of garlic extract on TNF-α expression and oxidative stress status in the kidneys of rats with STZ + nicotinamide-induced diabetes

**DOI:** 10.1080/13880209.2016.1255978

**Published:** 2016-12-09

**Authors:** Nasrin Ziamajidi, Abolfazl Nasiri, Roghayeh Abbasalipourkabir, Somayeh Sadeghi Moheb

**Affiliations:** a Department of Clinical Biochemistry, School of Medicine, Hamadan University of Medical Sciences, Hamadan, Iran;; b Students Research Center, Hamadan University of Medical Sciences, Hamadan, Iran

**Keywords:** *Allium sativum*, tumour necrosis factor-alpha, diabetic nephropathy

## Abstract

**Context:***Allium sativum* L. (Liliaceae) (garlic) is a medicinal plant that is widely used in herbal medicine. Nephropathy is a complication of diabetes that is induced by long-term hyperglycaemia.

**Objective**: The effects of aqueous extract of garlic (AGE) on the expression of tumour necrosis factor-alpha (TNF-α) and oxidative stress status were studied in the kidneys of rats with streptozotocin (STZ) + nicotinamide-induced diabetes.

**Materials and methods:** Twenty-four Wistar rats were divided into four groups: control rats, rats with STZ + nicotinamide-induced diabetes that received a single dose of STZ (65 mg/kg) and nicotinamide (110 mg/kg) intraperitoneally, diabetic rats that were treated with garlic (2 g/kg/d, gavage), and normal rats that received garlic (2 g/kg/d, gavage). The glucose level was determined in the start of study, 7 d after induction of diabetes and 33 d after treatment with garlic. At the end of the treatment period, urea, uric acid and creatinine levels were estimated in sera. Malondialdehyde (MDA), total oxidant status (TOS), nitric oxide (NO) levels and TNF-α gene and protein expression were measured in the renal tissues of the rats.

**Results:** The glucose, uric acid, and urea levels increased in the serum of diabetic rats compared with control rats, and decreased in garlic-treated diabetic rats compared with diabetic rats (*p* < 0.05). MDA, TOS and NO increased (*p* < 0.001) in diabetic rats compared with the control group, and decreased in garlic-treated diabetic rats compared with diabetic rats (*p* < 0.01). The level of TNF-α mRNA did not differ between groups but the TNF-α protein level in diabetic rats was higher than in the control rats (*p* < 0.01), whereas after treatment with garlic, it was close to the normal level (*p* < 0.01).

**Discussion and conclusion:** These results indicate that garlic extract has hypoglycaemic, antioxidant and anti-inflammatory properties; therefore, it can be useful for the alleviation of diabetic complications.

## Introduction

Diabetic nephropathy is a most important complication of diabetes that causes the end-stage renal disease in many diabetic patients. The exact reason of diabetic nephropathy is unknown, but hyperglycaemia, advanced glycation products and activation of cytokines are proposed mechanisms for this complication (Brownlee 2001). The high blood glucose levels lead to induction of oxidative stress by increase the production of reactive oxygen species (ROS), promote lipid peroxidation, loss of function of different cell types such as renal cells (Diaz-Flores et al. 2012). Hyperglycaemia and oxidative stress activate immune system and create inflammatory medium by activation of the nuclear transcription factors-kappa B (NF-κB), and release of inflammatory cytokines, such as tumour necrosis factor-alpha (TNF-α) (Elmarakby & Sullivan 2012).

Herbal medicine by using the plants extract was commonly used before the development of modern medicines, but they are still widely used in many countries for treatment of common disease (Nasiri et al. 2014; Behrouj et al. 2015). One of the most conventional plants in herbal medicine is *Allium sativum* L. (Liliaceae) (garlic), which is used for the treatment of various diseases such as diabetes mellitus (Liu et al. 2007).

Therefore, the aim of the present study was to evaluate the possible therapeutic effects of aqueous extract of garlic on streptozotocin (STZ)+nicotinamide-induced diabetes in rats through studying TNF-α expression and oxidative stress status in the kidney tissues.

## Materials and methods

### Preparation of aqueous garlic extract (AGE)

Fresh garlic bulbs were purchased from local market in Hamadan, Iran. The plant was taxonomically identified by botanists in the herbarium department of biology, Bu-Ali Sina University, Hamadan, Iran. The cloves were peeled, washed and cut into small pieces. About 50 g was blended in 250 mL of distilled water, and homogenized in a mixing machine. The supernatant was filtered through Whatman No. 1 filter paper. Garlic extract was used freshly or quickly frozen until used. Daily 1 mL of this solution/100 g body weight (∼2 g/kg) was given to the rats by gavage (El-Demerdash et al. 2005).

### Animals and experimental design

Adult male Wistar rats weighing around 250–300 g (6–8 week old) were obtained from Hamadan University of Medical Sciences, Hamadan, Iran. The animals housed in standard cages with 12 h light-dark cycles, constant temperature of 25 ± 2 °C and free access to food and water. The animals were acclimatized for at least 5 d under these conditions before the start of the experiments. The rats were divided randomly into four groups each comprising of six animals. For induction of diabetes combination of STZ (Sigma, St. Louis, MO) and nicotinamide (Sigma, St. Louis, MO) were used.

Group 1 (control): normal rats that received single dose of citrate buffer (0.1 M, pH 4.5).

Group 2 (DM): Rats that received single dose of STZ (65 mg/kg body weight, intraperitoneally) dissolved in freshly prepared cold citrate buffer (0.1 M, pH 4.5) 15 min after the injection of nicotinamide (110 mg/kg, intraperitoneally) for induction of DM (Mojani et al. 2014). To prove the induction of diabetes, 7 d after injection of STZ and nicotinamide, blood glucose was measured using a strip operated blood glucose sensor (Accuchek, Roche, Waiblingen, Germany). Animals were considered diabetics if their fasting blood glucose concentrations exceed 250 mg/dL.

Group 3 (DM + AGE): diabetic rats that (similar to group 2) treated with AGE (2 g/kg body weight/day, gavage, 33 d).

Group 4 (AGE): normal rats that received AGE (2 g/kg body weight/d, gavage, 33 d).

At the end of treatment period, serum samples were collected from all groups and stored at −20 °C for measuring biochemical parameters. The kidneys were snap-frozen in liquid nitrogen and stored at −80 °C for the determination of oxidative stress status, nitrite level and expression level of TNF-α mRNA and protein. A small portion of kidneys was fixed in 10% formalin for histological evaluation.

### Determination of biochemical parameters

Biochemical parameters such as glucose, urea, creatinine and uric acid were measured in the serum of rats using commercially available kits (Pars Azmoon Diagnostics, Iran).

### Preparation of kidney tissue homogenate

Kidney tissue homogenates were prepared on ice using lysis buffer (10 mM KCl, 1.5 mM MgCl_2_, 1 mM EDTA, 0.1% Triton X-100, 10 mM HEPES, 0.5 mM DTT, protease inhibitor cocktail, pH 7.9) and incubated on ice for 20 min. The protein concentrations of kidney homogenate samples were determined by using Bradford method (Bradford 1976).

### Determination of oxidative stress status

Lipid peroxidation in kidney tissue homogenates was determined by malondialdehyde (MDA) assay according to Yagi method. The results were expressed as μmol MDA/mg protein content of the samples (Ohkawa et al. 1979).

Total oxidative status (TOS) in kidney tissue homogenates was determined using the oxidation of ferrous ion to ferric ion. The ferric ions form a coloured complex with xylenol orange in an acidic medium. Therefore, the colour intensity is related to the total number of oxidant molecules present in the sample. The results were expressed as μmol TOS/mg protein content of the samples (Erel 2005).

### Determination of nirtic oxide (NO)

The kidney NO level was determined by measuring the nitrite concentration in the kidney tissue homogenates using Griess method. The results were expressed as μmol nitrite/mg protein content of the samples (Miranda et al. 2001).

### Determination of TNF-α gene expression by real-time PCR

Total RNA was extracted from the kidney tissues of control and experimental samples using the RNX-Plus solution (Sinaclon, Tehran, Iran) according to the instructions of the manufacturer. The purified RNA was quantified by spectrophotometer (A_260_) and its quality was examined by electrophoresis. RNA (1 μg) was reverse transcribed to synthesize cDNA using RevertAid First Strand cDNA Synthesis kit (Thermo Scientific, Vilnius, Lithuania) according to the instructions of the manufacturer. Quantitative real-time PCR was performed on cDNA samples using the SYBR Premix ExTaq real-time PCR kit (Takara Bio Inc, Tokyo, Japan), according to the protocols of the manufacturer. Primer sequences were as follows: TNF-α (forward), GTCGTAGCAAACCACCAAGC, (reverse), CTCCTGGTATGAAATGGCAAA, 18s rRNA (forward), GTAACCCGTTGAACCCCATT, (reverse), CCATCCAATCGGTAGTAGCG. The relative changes in gene expression were determined using 2^−ΔΔCT^ method (Livak & Schmittgen 2001).

### Determination of TNF-α protein

The level of TNF-α in kidney tissue homogenates was evaluated by using rat TNF-α Platinum ELISA kit (eBioscience Bender MedSystems, Vienna, Austria) according to the instruction of the manufacturer.

### Histological study

In the end of treatment time, the kidney tissues of rats were quickly removed and a portion of their fixed in 10% formalin for a week at room temperature. The specimens were dehydrated in ethanol, cleared in xylene, embedded in paraffin, sectioned by microtome and finally stained with haematoxylin and eosin (H&E).

### Statistical analysis

Statistical analysis was performed using the SPSS software version 16 (SPSS, Chicago, IL) and analysis of variance (ANOVA) was used to compare means in different groups. Data were reported as mean ± standard deviation (SD) and significance was taken at *p* < 0.05.

## Results

### Effects of AGE on body weights in duration of treatment

The effects of AGE on the body weights are shown in Table 1. In the start of study, the 10th, 20th and 30th days of the study body weights of rats did not different significant in any groups. In the 10th and 30th days of study, the body weight of rats was decreased in the DM group in comparison with control rats but was not significant. In the 40th day of study body weights were significantly (*p* < 0.01) decreased in DM rats compared with control rats and significantly (*p* < 0.05) increased in the DM + AGE group compared with DM rats.

**Table 1.** Body weights in control rats, diabetic rats (DM), diabetic rats that treated with garlic extract (DM + AGE) and normal rats that received garlic extract (AGE).

	Body weight (g)				
Groups	First day	10th day	20th day	30th day	40th day
Control	266.33 ± 10.08	269.5 ± 9.7	290.17 ± 11.43	288.5 ± 8.71	309.17 ± 11.93
DM	271.17 ± 17.55	264.17 ± 22.22	291.5 ± 54.5	272.83 ± 28.37	265 ± 21.39**
DM + AGE	266.5 ± 8.93	265.17 ± 8.68	280.5 ± 17.62	288 ± 24.55	301 ± 25.95†
AGE	285.33 ± 12.87	286.17 ± 12.35	282.83 ± 11.82	294.4 ± 17.78	301.6 ± 13.86

Results are mean ± SD (*n* = 6). **p < 0.01 compare with control; †*p* < 0.05 compare with DM.

### Effects of AGE on glucose levels

Table 2 shows the effects of AGE on glucose levels. In diabetic rats, glucose level was increased significantly (*p* < 0.001) when compared with normal control rats. Oral administration of AGE in DM rats caused significant (*p* < 0.05) decrease in glucose level compared with untreated diabetic rats. This finding showed hypoglycaemic effect of aqueous garlic extract.

**Table 2.** Glucose levels in control rats, diabetic rats (DM), diabetic rats that treated with garlic extract (DM + AGE) and normal rats that received garlic extract (AGE).

	Glucose levels (mg/dL)		
Groups	Before induction of DM	7 d After induction of DM	End of treatment
Control	86.66 ± 6.97	85.66 ± 8.83	89.12 ± 10.8
DM	86.5 ± 6.11	367 ± 102.25***	327 ± 140.31***
DM + AGE	84.5 ± 3.65	345.8 ± 19.04	200 ± 74.3†
AGE	86.6 ± 3.09	83.66 ± 3.44	90.29 ± 10.1

Results are mean ± SD (*n* = 6). ***p < 0.001 compare with control; †*p* < 0.05 compare with DM.

### Effects of AGE on urea, creatinine and uric acid levels

In Table 3, the levels of urea, creatinine and uric acid in four groups of rats are summarized. The level of creatinine was not different between groups but urea and uric acid concentrations significantly (*p* < 0.05) increased in DM rats compared with control rats. Oral administration of AGE decreased significantly the level of urea (*p* < 0.01) and uric acid (*p* < 0.05) in the DM + AGE group compared with DM.

**Table 3.** Urea, creatinine and uric acid levels in control rats, diabetic rats (DM), diabetic rats that treated with garlic extract (DM + AGE) and normal rats that received garlic extract (AGE) at the end of experiment time.

Groups	Urea (mg/dL)	Creatinine (mg/dL)	Uric acid (mg/dL)
Control	53.6 ± 6.31	0.67 ± 0.06	1.89 ± 0.39
DM	93.16 ± 42.36*	0.68 ± 0.04	3.32 ± 1.62*
DM + AGE	42.66 ± 9.07††	0.78 ± 0.05	2.07 ± 0.45†
AGE	56 ± 5.61	0.75 ± 0.06	2.29 ± 0.47

Results are mean ± SD (*n* = 6). *p < 0.05 compare with control; †*p* < 0.05 and ††*p* < 0.01 compare with DM.

### Effects of AGE on oxidative stress status in kidney tissues

**Figure 1.**
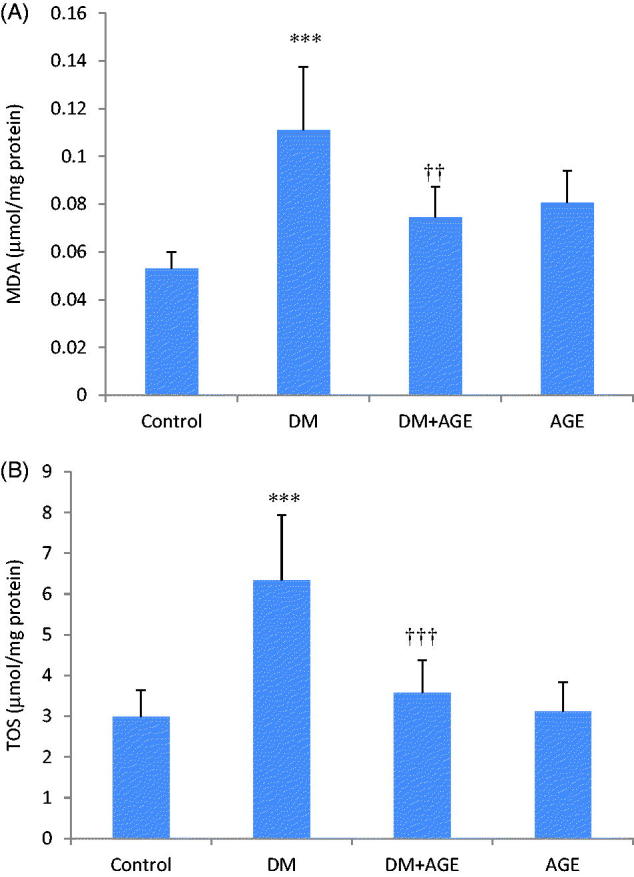
(A) Malondialdehyde (MDA) and (B) total oxidative status (TOS) levels in the kidney tissues of control rats, diabetic rats (DM), diabetic rats that treated with garlic extract (DM + AGE) and normal rats that received garlic extract (AGE). Results are mean ± SD (*n* = 6). ***p < 0.001 compare with control; ††*p* < 0.01 and †††*p* < 0.001 compare with DM.

The level of MDA in kidney tissue homogenates was increased significantly (*p* < 0.001) in the DM group compared with the control group and decreased significantly (*p* < 0.01) in the DM + AGE group compared with the DM group (Figure 1A).

The level of TOS in kidney tissue homogenates was increased significantly (*p* < 0.001) in the DM group compared with the control group and decreased significantly (*p* < 0.001) in the DM + AGE group compared with the DM group (Figure 1B).

### Effects of AGE on NO level of kidney tissues

**Figure 2.**
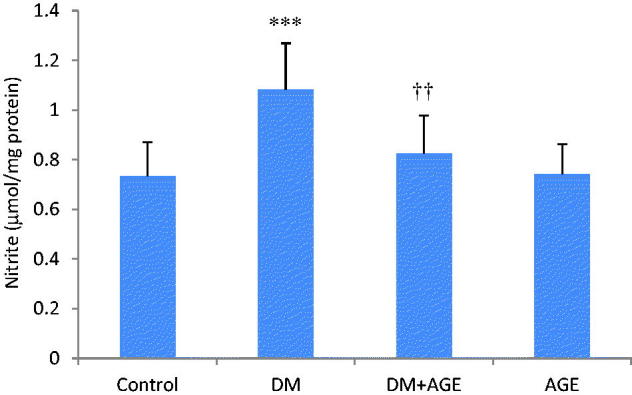
Nitrite levels in the kidney tissues of control rats, diabetic rats (DM), diabetic rats that treated with garlic extract (DM + AGE) and normal rats that received garlic extract (AGE). Results are mean ± SD (*n* = 6). ***p < 0.001 compare with control; ††*p* < 0.01 compare with DM.

As a parameter of NO synthesis nitrite concentration was assessed. The nitrite level in the kidney tissues of the DM group was elevated significantly (*p* < 0.001) compared with the control group and decreased significantly (*p* < 0.01) in diabetic rats treated with AGE comparison with the DM group (Figure 2).

### Effects of AGE on mRNA folding changes of TNF-α in kidney tissues

**Figure 3.**
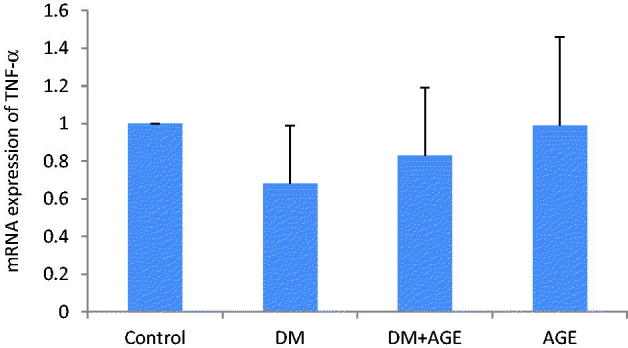
mRNA changes of tumour necrosis factor-alpha (TNF-α) in the kidney tissues of control rats, diabetic rats (DM), diabetic rats that treated with garlic extract (DM + AGE) and normal rats that received garlic extract (AGE). Results are mean ± SD (*n* = 6).

The mRNA levels of TNF-α in kidney tissues is shown in Figure 3. The expression of TNF-α was unaffected in the DM group compared with control group and in DM + AGE group compared to diabetic rats.

### Effects of AGE on TNF-α protein level

**Figure 4.**
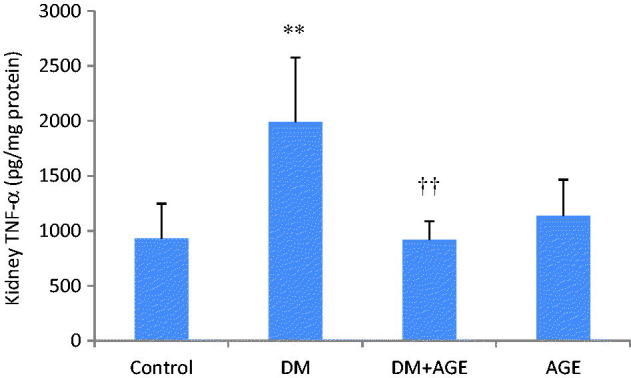
(A) Tumour necrosis factor-alpha (TNF-α) protein levels in the kidney tissues of control rats, diabetic rats (DM), diabetic rats that treated with garlic extract (DM + AGE) and normal rats that received garlic extract (AGE). Results are mean ± SD (*n* = 6). **p < 0.01 compare with control; ††*p* < 0.01 compare with DM.

The level of TNF-α protein in kidney tissue homogenates was increased significantly (*p* < 0.01) in DM group compared with control group and decreased significantly (*p* < 0.01) in DM + AGE group compared with DM group (Figure 4).

### Effects of AGE on histopathology of kidneys

**Figure 5.**
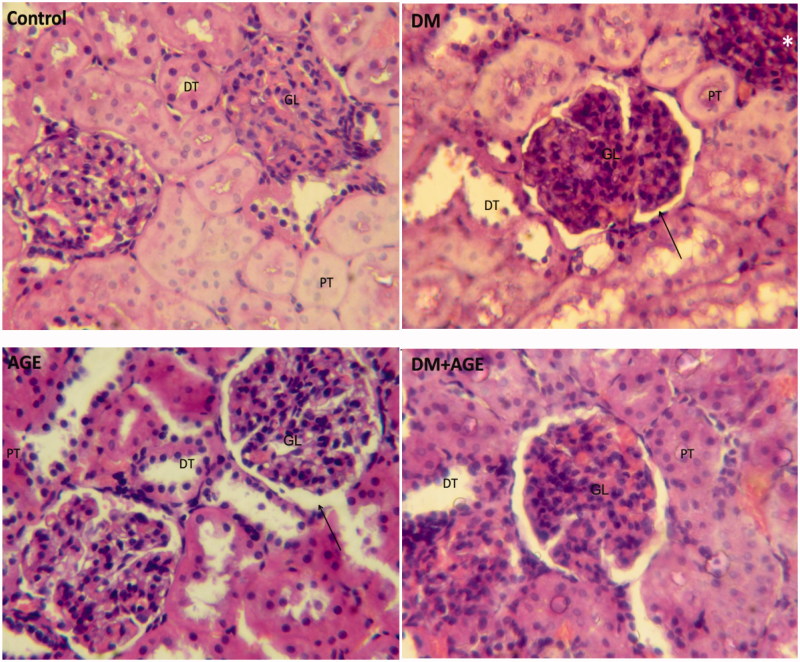
Histological staining with H&E in the kidney tissues of control rats, diabetic rats (DM), diabetic rats that treated with garlic extract (DM + AGE) and normal rats that received garlic extract (AGE). GL: glomerulus, PT: proximal tube, DT: distal tube. *Interstitial inflammation and → Bowman capsule space.

The kidney tissues photomicrographs of control and experimental groups of rats are shown in Figure 5. In control rats, glomerulus and Bowman capsule space were normal and proximal tube with normal columnar epithelium and distal tube with cubic epithelium were seen.

In diabetic animals (DM), proximal and distal tubes were expanded. Glomerulus size was normal and similar to control rats but Bowman capsule space was increased. Also interstitial inflammation was shown in these rats. In diabetic rats treated with garlic (DM + AGE) general structure was improved and the expansion of proximal and distal tubes was decreased whereas expansion of Bowman capsule space was not affected. In garlic-treated normal rats (AGE), a little expansion in proximal and distal tubes was seen but glomerulus size was normal.

## Discussion

Diabetes mellitus induced by STZ and nicotinamide in animals is considered a good model for the study of hyperglycaemia (Ziamajidi et al. 2013). STZ causes alkylation and degradation of DNA in β cells of the pancreas and impairs insulin secretion, whereas nicotinamide partly protects β cells from toxicity of STZ and causes induction of a milder form of diabetes mellitus (Mojani et al. 2014). Reduction of insulin will cause hyperglycaemia as has been proven in many studies (Rezagholizadeh et al. 2016). In this research, similar to other studies, the glucose level increased after induction of diabetes by STZ and nicotinamide (Shirwaikar et al. 2006). In diabetic rats, the body mass and weight of rats decrease as a result of insulin deficiency, and reduction in glucose transport into cells such as muscles (Liu et al. 2007). We showed this decrement in body weight in diabetic rats.

Garlic extract is a common herbal plant that is used in many cultures for treatment of different diseases (Arivazhagan et al. 2000). One of the most widespread diseases in the world is diabetes mellitus. The current study demonstrated that oral administration of garlic extract caused a decrease in the glucose level, and an increase in body weight in diabetic rats. This finding confirmed the improving effect of garlic on glucose tolerance (El-Demerdash et al. 2005; Eidi et al. 2006).

The majority of diabetic patients suffer from nephropathy, which is a major complication of diabetes. In this condition, urea, uric acid and creatinine, as markers of kidney function, increase in serum. Kidney histopathology shows important differences in the structure and arrangement of proximal and distal tubes (Sivakumar et al. 2010). Natural or chemical drugs have improvement effects on renal function markers and histopathology of this organ (Gui et al. 2013). The results of this study suggest that aqueous garlic extract has the opposite effect on the renal function markers and histopathology of diabetic rats. Since hyperglycaemia causes the diabetic complications, compounds that have hypoglycaemic effects can be effective in reducing of diabetic complications such as renal dysfunction.

Considerable evidence in experimental and clinical studies proposes a link between a high level of glucose in the serum, oxidative stress, and diabetic complications. Hyperglycaemia causes the increase of electron flow in mitochondria. It also inhibits the complex III and increases production of superoxide free radicals. In this condition, the balance between production of free radicals and antioxidant defence is impaired. Free radicals attack several molecules such as lipids, proteins, and DNA (Du et al. 2000). So, lipid peroxidation (MDA) and TOS as indicators of oxidative stress are measured. In diabetic animals, MDA and TOS levels increased significantly as a result of hyperglycaemia and oxidative stress induction. Garlic as a medicinal plant has antioxidant capacity. It contains antioxidant compounds such as *S*-allyl cysteine, which scavenge free radicals (Saravanan & Ponmurugan 2011). Therefore, the MDA and TOS levels decreased in diabetic rats that were treated with oral administration of garlic extract.

Many researchers believe that diabetes mellitus is an inflammatory disease because the concentration of cytokines such as interleukin (IL)-6, IL-18, IL-1 and TNF-α is elevated in the serum of these patients (Ingaramo et al. 2011). Hyperglycaemia and oxidative stress cause activation of NF-κB and activator protein-1 (AP-1), and lead to the transcription of cytokines and growth factor genes. TNF-α, IL-1 and IL-8 are cytokines that are up-regulated by activation of NF-κB. Moreover, in a diabetic condition, macrophage migration induces the release of cytokines, which stimulates oxidative stress induction. Therefore, this condition produces a vicious cycle and increases the damage of renal cells (Elmarakby & Sullivan 2012). Growth factors such as transforming growth factor-β (TGF-β) and vascular endothelial growth factor (VEGF) are hypertrophic and fibrogenic agents that up-regulate by oxidative stress and contribute to cellular hypertrophy and enhanced collagen synthesis as well as to vascular changes in diabetic nephropathy (Ziyadeh 2008).

One of the target genes for NF-κB is inducible nitric oxide synthase (iNOS). Oxidative stress and hyperglycaemia cause activation of NF-κB and an increase in the expression of the iNOS gene and production of nitric oxide (NO). Elevation of NO results in the starting cascade of the apoptosis pathway, which can impair normal tissue functions and their histology (Ingaramo et al. 2011). In many studies, nitrite concentration was measured to determine the NO level (Dirsch et al. 1998). Garlic extract decreased the nitrite level. Consequently, it can be useful in kidney dysfunction.

## Conclusions

In conclusion, aqueous extract of garlic as a natural component due to its hypoglycaemic and antioxidant properties may lead to improvements in complications of diabetes such as renal dysfunction.
